# A systematic review and meta-analysis on chloroquine and hydroxychloroquine as monotherapy or combined with azithromycin in COVID-19 treatment

**DOI:** 10.1038/s41598-020-77748-x

**Published:** 2020-12-17

**Authors:** Ramy Mohamed Ghazy, Abdallah Almaghraby, Ramy Shaaban, Ahmed Kamal, Hatem Beshir, Amr Moursi, Ahmed Ramadan, Sarah Hamed N. Taha

**Affiliations:** 1 Tropical Health Department, High Institute of Public Health, Alexandria University, Alexandria, Egypt; 2 Department of Cardiology, Faculty of Medicine, Alexandria University, Alexandria, Egypt; 3 Department of Instructional Technology and Learning Sciences, Utah State University, Logan, USA; 4 Hepatology Unit, Department of Internal Medicine, Faculty of Medicine, Alexandria University, Alexandria, Egypt; 5 Department of Cardiothoracic Surgery, Faculty of Medicine, Mansoura University, Mansoura, Egypt; 6 Department of Cardiothoracic Surgery, Amreya General Hospital, Egyptian Ministry of Health and Population, Alexandria, Egypt; 7 Department of Neurosurgery, NHS Tayside Trust, London, UK; 8 Department of Medical Information and Data Science, DataClin CRO, Cairo, Egypt; 9 Department of Forensic Medicine and Clinical Toxicology, Faculty of Medicine, Cairo University, Cairo, Egypt

**Keywords:** Diseases, Infectious diseases, Viral infection

## Abstract

Many recent studies have investigated the role of either Chloroquine (CQ) or Hydroxychloroquine (HCQ) alone or in combination with azithromycin (AZM) in the management of the emerging coronavirus. This systematic review and meta-analysis of either published or preprint observational studies or randomized control trials (RCT) aimed to assess mortality rate, duration of hospital stay, need for mechanical ventilation (MV), virologic cure rate (VQR), time to a negative viral polymerase chain reaction (PCR), radiological progression, experiencing drug side effects, and clinical worsening. A search of the online database through June 2020 was performed and examined the reference lists of pertinent articles for in-vivo studies only. Pooled relative risks (RRs), standard mean differences of 95% confidence intervals (CIs) were calculated with the random-effects model. Mortality was not different between the standard care (SC) and HCQ groups (RR = 0.99, 95% CI 0.61–1.59, I^2^ = 82%), meta-regression analysis proved that mortality was significantly different across the studies from different countries. However, mortality among the HCQ + AZM was significantly higher than among the SC (RR = 1.8, 95% CI 1.19–2.27, I^2^ = 70%). The duration of hospital stay in days was shorter in the SC in comparison with the HCQ group (standard mean difference = 0.57, 95% CI 0.20–0.94, I^2^ = 92%), or the HCQ + AZM (standard mean difference = 0.77, 95% CI 0.46–1.08, I^2^ = 81). Overall VQR, and that at days 4, 10, and 14 among patients exposed to HCQ did not differ significantly from the SC [(RR = 0.92, 95% CI 0.69–1.23, I^2^ = 67%), (RR = 1.11, 95% CI 0.26–4.69, I^2^ = 85%), (RR = 1.21, 95% CI 0.70–2.01, I^2^ = 95%), and (RR = 0.98, 95% CI 0.76–1.27, I^2^ = 85% )] respectively. Exposure to HCQ + AZM did not improve the VQR as well (RR = 3.23, 95% CI 0.70–14.97, I^2^ = 58%). The need for MV was not significantly different between the SC and HCQ (RR = 1.5, 95% CI 0.78–2.89, I^2^ = 81%), or HCQ + AZM (RR = 1.27, 95% CI 0.7–2.13, I^2^ = 88%). Side effects were more reported in the HCQ group than in the SC (RR = 3.14, 95% CI 1.58–6.24, I^2^ = 0). Radiological improvement and clinical worsening were not statistically different between HCQ and SC [(RR = 1.11, 95% CI 0.74–1.65, I^2^ = 45%) and (RR = 1.28, 95% CI 0.33–4.99), I^2^ = 54%] respectively. Despite the scarcity of published data of good quality, the effectiveness and safety of either HCQ alone or in combination with AZM in treating COVID-19 cannot be assured. Future high-quality RCTs need to be carried out.

**PROSPERO registration**: CRD42020192084.

## Introduction

Coronavirus disease-2019 (COVID-19) is a serious health problem caused by the novel Coronavirus (nCOV) or Severe Acute Respiratory Syndrome Coronavirus 2 (SARS-CoV-2)^1^. SARS-COV-2 is a member of the Coronavirus family, a family that was previously responsible for Severe Acute Respiratory Syndrome (SARS) in 2002 and Middle East Respiratory Syndrome (MERS) in 2012^2^. COVID-19 was emerged by the end of 2019 at Wuhan City in China and was notified by WHO to be a pandemic in March 2020^3^. Till the 19th of July, 2020, around 14 million COVID-19 cases and 605,225 deaths were reported worldwide^4^.

Till now, there is no effective treatment for COVID-19^5^. Chloroquine (CQ) was initially reported to be effective against SARS-COV-2 and then Hydroxychloroquine (HCQ) followed^6^. SARS-COV-2 is known to bind to human cells via the Angiotensin-Converting Enzyme 2 (ACE 2) receptor^7,8^. In-vitro studies showed that CQ and HCQ cause glycosylation of ACE2 receptor making cells to be refractory to SARS-COV-2 infection^8^. This makes the drugs possible players in the treatment and even the prophylaxis against COVID-19.

Both drugs have also been shown to have immunomodulatory effects^9^. HCQ is now broadly used in autoimmune diseases such as systemic lupus erythematosus (SLE) and rheumatoid arthritis (RA)^9^. This makes both drugs potentially effective in reducing the severity of COVID-19 through suppressing the immune system response to SARS-COV-2, which is now thought to be at least partly responsible for the severe forms of the disease^8^.

The safety of both drugs is also an important issue. Although both drugs are generally well-tolerated, high doses can be associated with severe side effects like myopathy, neuropathy, and cardiomyopathy^10^. Retinopathy is a well-known side effect that is related to drug-prolonged use^9^. Usage of CQ or HCQ in critically ill patients can carry a higher risk of side effects, especially when combined with other drugs that carry a risk of QT interval prolongation increasing the risk of torsade’s de points^11,12^. In the elderly, HCQ was found to increase the risk of drug-drug interaction, which may lead to patients’ ineligibility to participate in COVID-19 HCQ trials^13^.

In-vivo studies showed contradictory results regarding CQ and HCQ in COVID-19. Firstly, Chinese researchers reported the efficacy of CQ against COVID-19^14^. Then, a French research group reported the efficacy of HCQ added to Azithromycin (AZM) in decreasing viral load^15^. After that, many studies reported that these drugs had no benefit or even may cause harmful effects^12^. Here, we conducted an in-vivo systematic review and meta-analysis on the effectiveness and safety of either CQ or HCQ alone or in combination with AZM in treating COVID-19.

## Methods

We performed this systematic review in strict compliance with the preferred reporting items of the systematic review and meta-analysis (PRISMA) checklist^16^. All steps were conducted in concordance with the Cochrane Handbook of Systematic Review and Meta-Analysis^17^.

### Inclusion and exclusion criteria

#### Inclusion criteria

Studies satisfying the following criteria were included:

- Recruited patients with confirmed SARS-COV-2 virus confirmed by Polymerase Chain Reaction (PCR).
- Declared the effect of CQ or HCQ as anti-SARS-COV-2.
- Had a comparator group receiving either standard care or placebo.
- No restriction regarding country, race, gender, or age.

#### Exclusion criteria

Any study had one of the following criteria were excluded:

- Published before 2019.
- Conducted in non-human subjects (in-vitro studies).
- Abstract-only papers as preceding papers, conference, editorial, and author response and books.
- Studies with data not reliably extracted, duplicate, or overlapping data.
- Any study written in any language other than English, French, or Chinese.
- Case report, case series, and systematic review studies.

#### Outcome

*Primary outcome*:

- Mortality of HCQ or CQ alone or in combination with AZM versus SC.

*Secondary outcome*:

- Hospital stay or number of days’ till discharge.
- Virological cure (proportion of virological cure either overall or at a certain time day 4, 10, or 14).
- The number of days till virological clearance.
- Need for mechanical ventilation (MV).

Other outcomes

- Clinical improvement: the resolution of cough or fever.
- Laboratory test improvement (serum ferritin, lymphocyte count).
- Radiological improvement.
- Safety of CQ and HCQ; reporting side effects and QT prolongation.

#### Comparisons

- HCQ or CQ in comparison to SC.
- HCQ + AZM in comparison to SC.

#### Outcomes conceptualization

*Mortality*: The primary outcome measured in this study was mortality which was defined as the percentage of deaths that occurred during a study period.

*Virological cure rate:* Defined as the proportion of patients who achieved negative PCR. Time to negative PCR: Defined as the number of days until the PCR becames negative. It also included the virological cure rate in the days matched between at least two studies. Based on the results, we found matching on days 4, 10, and 14.

*Hospital Stay*: was defined as the duration of patients' hospital stays measured in days.

*Radiological progression*: included the number of patients who showed progression in their radiological CT results during the period of a study.

*Clinical worsening*: was defined as the deterioration of the case during the study’s period, or development of complications such as severity progression, or worsening of clinical symptoms.

*Need for mechanical ventilation (MV)*: was represented by the percentage of patients who needed respiratory support through MV during treatment.

*Experiencing side effects*: This outcome included any side effect that happened from using the studied treatment during a study.

*QT prolongation:* In this study, we target specifically the effect of HCQ/CQ/AZM on QT prolongation during a study period which is defined as being more than 460 msec in females or 460 msec in males^10^.

### Data extraction

A computer literature searches of (PubMed, Google Scholar, Cochrane, Scopus, Web of Science, Segle, VLH, COVID-Inato, COVID-Trial, and Clinical Trial.gov) was conducted till June 5th, 2020 using the following keywords (Chloroquine OR Hydroxychloroquine) AND (2019 novel Coronavirus disease OR COVID-19 OR SARS-CoV-2 OR novel Coronavirus infection OR 2019-ncov infection OR Coronavirus disease 2019 OR Coronavirus disease-19 OR 2019-ncov disease OR COV OR Coronavirus). (Eight independent reviewers screened the literature search result for relevant studies according to the pre-specified inclusion and exclusion criteria.

All records were exported to an Endnote library to detect and remove duplicates using the “remove duplicate function”. All references that had the same title and author and published in the same year or the same journal were removed. References remaining after this step were exported to a Microsoft Excel file with essential information for screening.

The title and abstract screening were done by seven independent reviewers to select papers based on the inclusion criteria. Each article was checked by two independent reviewers. Any disagreement was solved by the first author (RG). During the full-text screening phase, all selected articles were downloaded, and the full text was reviewed by two independent reviewers. The decision to include or exclude articles for qualitative and quantitative analysis should be agreed upon by the two reviewers to pass through. If any disagreement was noticed, the first author was asked to give his decision. The completed data were then thoroughly checked by two reviewers (RG, AK).

We applied three methods to do manual searching. Firstly, we searched the reference lists of the included articles. Secondly, we performed citation tracking in which the reviewers track all the articles that cite each one of the included articles. This might involve the electronic searching of databases. Thirdly, like citation tracking, we followed all “related to” or “similar “articles. All excluded records were given exclusion reasons. Manually added research included preprint, and unpublished data if fulfilling the inclusion criteria.

During the data extraction and the quality assessment, in a Microsoft Excel sheet, two reviewers extracted data related to patient characteristics and outcomes (authors, year of publication, country of patients, inclusion or exclusion criteria, when the study was conducted, study design, sample size, treatment option, dosage and duration, adverse events, primary and secondary outcomes).

### Data analysis

#### Method of data analysis

Data were analyzed using Review Manager Software V5.3 for Windows. For the continuous variables, data were pooled using the standardized mean difference (SSMD). For categorical variables, data were pooled using Risk Ratio (RR) with the perspective of a 95% Confidence Interval (CI) in the meta-analysis model. In the case of zero frequency, the correction value of 0.1 was used. In the case of significant heterogeneity, we used the random effect model, otherwise, the fixed-effect model was used. Meta-regression analysis was done to examine the impact of the age difference, disease severity, sex, and country on HCQ regimen group mortality RR.

#### Heterogeneity

Heterogeneity was assessed by the Chi-Square test (X^2^) and measured by the I-Square test. Following Cochrane Handbook for Systematic Reviews of Interventions 10, I^2^ was interpreted as follows: “0% to 40%: might not be important; 30% to 60%: may represent moderate heterogeneity; 50% to 90%: may represent substantial heterogeneity; 75% to 100%: considerable heterogeneity. The importance of the observed value of I^2^ depends on the magnitude and direction of effects, and strength of evidence for heterogeneity (e.g. *P *value from the chi-squared test, or a confidence interval for I^2^). In the case of heterogeneity, DerSimonian and Laird random-effects models were applied to pool the outcomes. Otherwise, the inverse variance fixed-effect model was used. Forest plots were presented to visualize the degree of variation between studies. In the case of missing standard deviation (SD), we calculated it from the corresponding 95% confidence interval or the standard error^18^. In the case of the absence of mean, authors were emailed and asked for the required data, or they were calculated according to the mathemetical equation developed by Wan, et al.^19^.

#### Quality assessment

Quality assessment (QA) of the research depended on the study design. The risk of bias in the individual studies included for meta-analysis was assessed using the Cochrane risk assessment tool in cases of randomized control trials (RCTs)^20^, study quality assessment tools for observational studies^21^, and Robins-1 for non-randomized control trial^22^. The assessment was performed by three independent reviewers (AA, AK, SH) and further checked by two additional reviewers (RG, RS).

### Sensitivity analysis

Sensitivity analysis is known to be an essential part of systematic reviews with meta-analyses to determine the robustness of the obtained outcomes to the assumptions made in the data analysis^23^. We conducted leave one out sensitivity analysis to examine the effect of studies that greatly influenced the result, especially by their weight through excluding them from the meta-analysis.

## Results

### Study selection process

**Figure 1:**
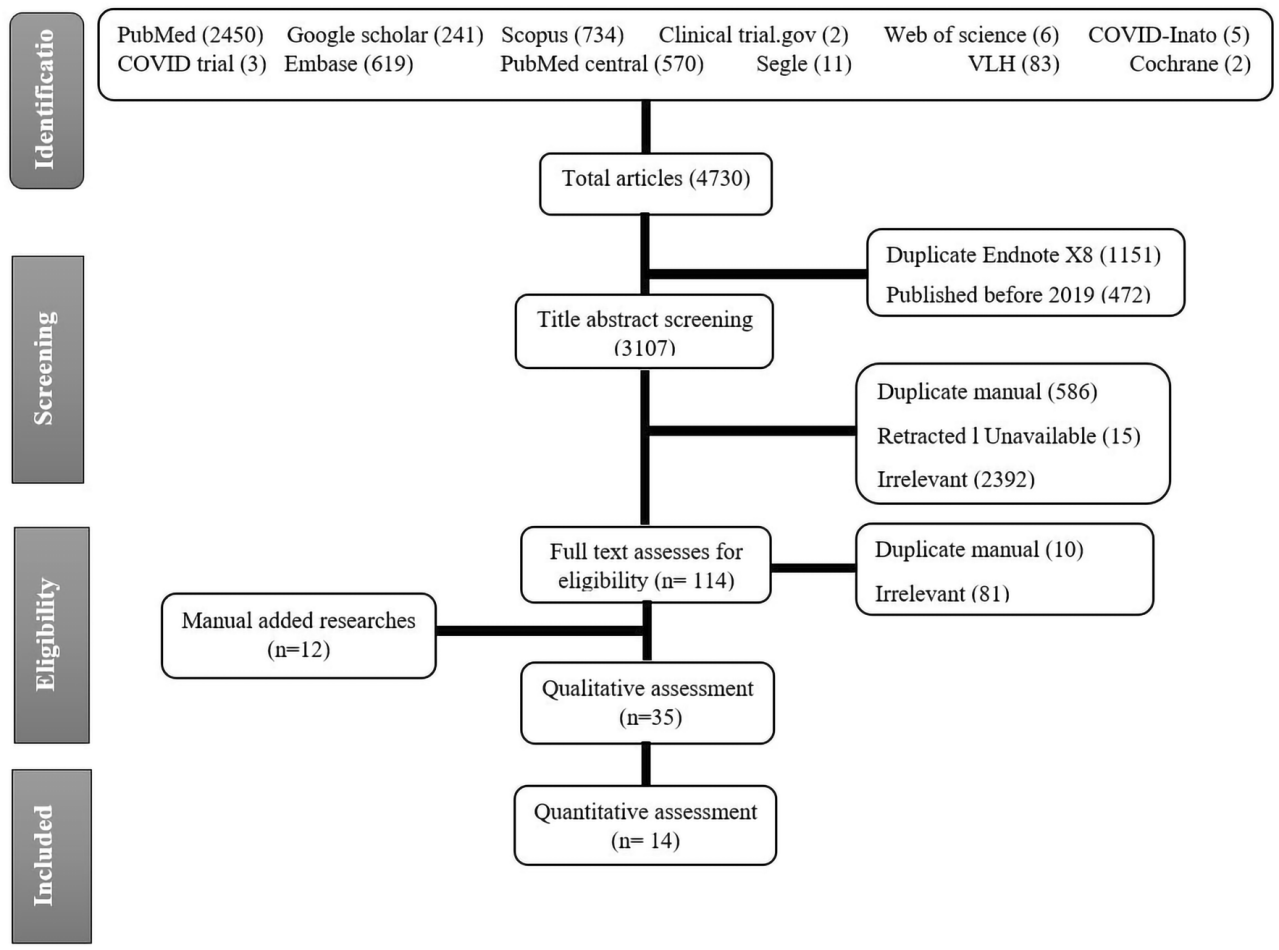
PRISMA flow chart of studies screened and included.

A total of 4730 articles were found after searching 12 different databases. Of this number, 1151 duplicates were found by Endnote X8, and 472 were published before 2019 so they were excluded. Title and abstract screening of 3107 papers resulted in exclusion of irrelevant papers (2394), retracted articles (15), and manually found duplicates (586). A total of 112 articles were screened for eligability. Finally, 23 papers were eligible, in addition to, 12 mannually added research. Of these 35 papers 14 studies entered in the meta-analysis (Fig. 1).

### Study characteristics

Fourteen studies were included in the meta-analysis: 3 RCTs, 2 non-RCT, 3 case–control studies, and 6 retro or prospective cohort studies. HCQ arms of the comparative studies have been combined with observational studies for effect size meta-analysis. The studies’ sample size ranged from 30 to 1438 participants. Characteristics of studies entered the systematic review presented in Table 1.

**Table 1:** All published studies that reported the effectiveness or safety of hydroxychloroquine, chloroquine, or azithromycin.

Study	Country	Case definition	No. patients (intervention/standard care)	^a^Age (intervention-standard care)	Sex (total male percentage)	Treatment	Duration (days)	Primary outcomes	Findings
	Type of study					Intervention			
	Study setting					Control			
Borba^25^	Brazil	Clincally suspected adults with severe COVID,	81 (41:40)	47.4 ± 13.3	75.3% males	HCQ 600 mg bid	23 March–5 April 2020	Lethality until day 13	39% (high dose) and 15% (low dose)
	RCT					HCQ 450 mg bid for one day then 450 mg/d for 4 days			
Chang^26^	USA	COVID-19 positive	117 (HCQ: 66 HCQ + AZM: 51)	60.2 ± 14.9	59.5% males	HCQ 400 mg bid for 1 d then 200 mg bid for 4 d	–	Assess QTc	32.1 ± 25.1 ms (HCQ) 35.7 ± 28.9 ms (HCQ + AZM), *P* = 0.66
	Prospective cohort					HCQ as before + AZM			
Chen^27^	China	PCR-confirmed COVID-19	30 (15:15)	HCQ: 50.5 ± 3.8; SC: 46.7 ± 3.6	HCQ: 60% males; SC: 80% males	HCQ 400 mg/d for 5 d	6 Feb–25 Feb 2020	PCR conversion One week after hospitalization	86.7% (HCQ) and 93.3%(SC)
	RCT					SC			
Chen^28^	China	Covid-19	62 (31:31)	HCQ: 44.1 ± 16.1	Intervention 45.2% males	HCQ 400 mg/d	4 Feb–28 Feb 2020	Absorption of pneumonia in CT	80.6% (HCQ) and 54.8% (SC) Shorter duration of cough and fever in HCQ group
	RCT			SC: 45.2 ± 14.7	Standard care 48.3% males	SC		Clinical improvement	Two patients experienced side effect (HCQ)
									Four paients deterirated in the (SC)
Chong^29^	China	Covid-19	11	51.55 ± 12.54	63.6% males	LPV/r 400/100 mg bid for 14 days + HCQ 400 mg bid day‐1 then 200 mg bid for 2‐5)	N/A	Developed QT prolongation	27.3% developed prolonged QTc
	Case-series								
Gautret^30^	France	PCR positive mildly infected Covid-19 patients	80	52.5 (42–62)	53.8% males	HCQ 200 mg tid for 10 d + AZM 500 mg for 1 d then 250 mg/d for 4 d	3–21 March 2020	Clinical course, viral clearance and hospital stay	Clinical course: 81.3% with favorable outcome
	Retrospective observational								Viral clearance: 93% had viral clearance at Day8
									Hospital stay: mean length of stay of 4.6 days
Gautret^30^	France	PCR confirmed COVID-19 patients	36 (HCQ: 14 HCQ + AZM: 6; SC: 16)	Total HCQ 51.2 ± 18.7 SC: 37.3 ± 24.0	Total HCQ: 45% males; SC: 37.5% males	HCQ 200 mg tid for 10 d	Early March-16 March	Virological cure	57.1% (HCQ), 100% (HCQ + AZM) and 12.5% (SC)
	Clinical trial					HCQ as before + AZM: loading 500 mg then 250 mg/d for 4 d			
						SC			
Geleris^6^	USA	All Hospitalized adult patients with positive COVID-19 infection	1376 (811:565)	–	HCQ: 58.4% male; SC: 54.3% males	HCQ 600 mg bid one day then 400 mg/d for 4 days	7 March–8 April 2020	Composite of time to intubation or death (time-to-event analysis)	No significant association between HCQ and intubation or death (hazard ratio, 1.04; 95% CI 0.82–1.32)
	Observational					SC			
Gerard^31^	France	Reports of cardiotoxicity associated with HCQ, CQ, AZM, or LPV/r use in COVID-19	120	64.3 ± 13.4	76.7% males	HCQ/CQ/AZM/LPV/r	–	Cardiac adverse drug reactions	86% (HCQ), 60% (AZM), 14%( LPV/r) and 2.5% (CQ)
	Survey								
Hraiech^32^	France	COVID-19 PCR positive ICU patients	45 (HCQ + AZM: 17, LPV/r: 13, SC: 15)	HCQ + AZM: 60 ± 17 LPV/r: 62 ± 13; SC: 60 ± 16	HCQ + AZM: 88% males; LPV/r: 69% males; SC: 73% males	HCQ 600 mg and AZM 500 mg then 250 mg/d	2 March–31 March 2020	Viral clearance at day 6 treatment	PCR was negative in 5/13 (38%) from the LPV/r group, 3/17 (1%) from the HCQ + AZM group and 2/15 (20%) from the control group
						LPV/r: 800 mg/d			
	Case control					SC			
Macías^33^	Spain Retrospective cohort	patients with autoimmune rheumatic diseases with confirmed or suspected COVID 19	722 (290:432)	HCQ: 56 (45–65); No HCQ: 58 (48–68)	HCQ: 16%; No HCQ: 21%	HCQ vs. no HCQ (for autoimmune disease)	27 Feb–16 April 2020	Incidence of COVID 19 in patients with autoimmune rheumatic diseases receiving vs. not receiving HCQ	5 cases (1.7%) in those on HCQ vs. 5 cases (1.2%) in those not on HCQ
Mahévas^34^	France	severe acute respiratory syndrome	173 (84/89)	HCQ: 59 (48–67); SC: 62 (54–69)	HCQ: 77% males; SC: 67% males	HCQ 600 mg/d	12 March–31 March 2020	the survival rate at day 21 without transfer to ICU	76% (HCQ) and 75% (SC)
	Comparative observational					SC			
Rosenberg^35^	USA	Lab confirmed COVID-19	1438 (HCQ + AZM: 735; HCQ 27; AZM: 211SC: 221)	HCQ + AZM: 61.4; HCQ: 65.5; AZM: 62.5; SC: 64	HCQ + AZ: 62; HCQ: 58.3; AZM: 63.5; SC: 49.8	HCQ + AZ	15 March–28 March 2020	Mortality	25.7% (HCQ + AZM), 19.9% (HCQ), 10% (AZM) and 12.7% (SC)
						HCQ			
	Retrospective cohort					AZ			
						SC			
Stroppa^36^	Italy	COVID-19, Cancer patients	56	71.64 ± 10.08	80% males in cancer patients, 48% males in Non-cancer patients	7 days or HCQ 400 mg/d alone or AV + HCQ	21-Feb 21, 2020 to March 18, 2020	Mortality	Of the 25 cancer patients, nine (36%) are dead and 16 (64%) are alive, with improvement from pneumonia, in the control group of patients hospitalized and treated with the same protocol in the same period, 16.13% are dead and 83.87% are alive *P* = 0.12
	Case–control		25 Cancer patients,31 non-cancer patients						
Broek^37^	Netherlands	Hospitalized and suspected with COVID-19	95 patients	65 (min18-max 91)	66.3% males	CQ 600 mg then 300 mg bid for 5 d	8–27 March 2020	Assess the degree of CQ induced QTc prolongation in hospitalized COVID-19 patients	22 patients (23%) had a QTc interval exceeding 500 ms
	Retrospective observational study								
Voisin^38^	France	Hospitalized patients with COVID-19 pneumonia	50 patients	68 (53–81)	55.2% males	HCQ 600 mg/d for 6 d + AZM 500 mg/d for 1 d then 250 mg/d for 2–5 d N/A	18 March–25 March 2020	Effect of HCQ + AZM combination on QTc in case of short term treatment of COVID 19	38 patients (76%) presented short term modifications of QTc (> 30 ms)
	Cohort								
Yu^39^	China	Confirmed COVID-19 in critically ill adult patients	550 (48/502)	HCQ: 68 (60–75); SC: 68 (59–77)	HCQ: 66.7% males; SC: 62.2% males	HCQ 200 mg bid (7–10 days)	1 Feb 2020 to 4 April, 2020	Mortality and inflammatory cytokines level	Mortality: 18.8% (HCQ) and 47.7% (SC)
	Retrospective cohort					SC			IL-6 reduced from 22.2 (8.3–118.9) pg/ml to 5.2 (3.0–23.4) pg/ml (HCQ) but no change in (SC)
Huang^40^	China	Confirmed COVID-19 patients	22 CQ: 10 LPV/r: 12	CQ: 41.5 (33.8–50.0) LPV/r: 53.0 (41.8–63.5)	CQ: 30% LPV/r: 50%	CQ 500 mg bid for 10 d	27 January 2020 to 15 Feb 2020	Virological cure, CT scan improvement and hospital discharge at day 14	Virological cure: 100% (HCQ) and 91.7% (LPV/r)
	Case–control					LPV/r 400/100 mg bid for 10 d			CT scan improvement: 100% (HCQ) and 75% (LPV/r)
									Hospital discharge: 100% (HCQ) and 50% (LPV/r)
Magagnoli^41^	USA	Lab confirmed COVID-19 hospitalized patients	807 ( HCQ: 198 HCQ + AZ: 214 SC: 395)	HCQ: 71 (62–76.8), HCQ + AZ: 68 (59–74); SC: 70 (59–77)	HCQ: 97%; HCQ + AZ: 95.3%; SC: 95.2%	HCQ 400 mg/d for 5 d	9 March 2020–29 April 2020	Mortality and mechanical ventilation	Mortality: HCQ aHR, 1.83; 95% CI 1.16–2.89; *P* = 0.009, but HCQ + AZM aHR, 1.31; 95% CI 0.80–2.15; *P* = 0.28. compared to SC
	Retrospective cohort					HCQ 422 mg/d + AZM for 5 d			Mechanical ventilation: HCQ aHR, 1.19; 95% CI 0.78–1.82; *P* = 0.42 but in the HC + AZ aHR, 1.09; 95% CI 0.72–1.66; *P* = 0.69, compared to SC
						SC			
Ramireddy^42^	USA	COVID-19 Confirmed/suspected patients	98 (AZM: 27–HCQ: 10–AZM + HCQ: 61)	62.3 ± 17	61% males	HCQ + AZM	1 February 2020 to 4 April, 2020	QT prolongation	Significant prolongation in men (12% of patients) reached critical QTc prolongation
	Case-series					HCQ 400 mg bid on day1 then 200 bid on days 2 to 5			Changes in QTc were highest with the combination group compared to either drug alone, with many-fold greater prolongation with the combination vs. AZM alone (17 ± 39 vs. 0.5 ± 40 ms, *P* = 0.07)
						AZM either 500 mg daily or 500 mg on day1 followed by 250 mg daily on days 2–5			
Barbosa^43^	USA	PCR positive COVID-19 patients	63 (32/31)	HCQ: 61.8 ± 15; SC: 63.7 ± 15.4	HCQ: 46.9% males; SC: 71% males	HCQ 400 mg bid for 1–2 days then 200–400 mg/d for 3–4 days	15 March 2020–31 March 2020	Mortality rate	12.9% (HCQ) and 3.13% (SC)
	Retrospective cohort					SC			
Mallat^44^	UAE	Hospitalized adult patients with confirmed SARS-CoV-2 infection	34 (23/11)	HCQ: 33 (31–48); SC: 41 (30–55)	HCQ: 73.9% males; SC: 72.7% males	HCQ 400 mg bid for 1 day, then 400 mg/d for 10 days SC	1 March–25 March 2020	The time to SARS-CoV-2 negativity	17 (13–21) days HCQ and 10 (4–13) days SC
	Retrospective cohort								
Huang^3^	China	Confirmed COVID-19 cases	373 (197/176)	CQ: 43.8 ± 13.1; SC: 45.6 ± 13.5	CQ: 49% males; SC: 45% males	CQ 500 mg/d	7 Feb-8 March 2020	Median Time to undetectable viral RNA	3 (3–5) CQ and 9 (6–12) SC
	Prospective Observational					SC			
Feng^45^	China	Confirmed COVID-19 cases	50 (25/25)	CQ: 51 (41–62); SC: 46 (38–67)	50.4% of males	CQ 500 mg bid	Jan 17–Feb. 28, 2020	1ry outcome: development of severe pneumonia	None of patients treated with CQ developed severe pneumonia, though without significance (difference, 12.0%; 95% CI − 3.5 to 30.0%; *P* = 0.074)
	Retrospective cohort					SC			
Mathian^46^	France	SLE with COVID-19	HCQ: 17	53.5 (26.6–69.2)	23.5% males	HCQ	29 March–6 April 2020	Clinical course	Admitted to hospital (82%); needed O2 therapy (64.7), ICU admission (41%) Respiratory complications: ARDS (29%) Respiratory failiure (65%) Pneumonia (76%) Acute renal failure (17.6%), hemodialysis (11.8%) Discharge (36%), Death (14%), remained in hospital (50%)
	Case series					N/A			
Tang^47^	China	patients hospitalized with PCR confirmed mild to moderate COVID–19	150 (75/75)	HCQ 48.0 ± 14.1; SC 44.1 ± 15.0	HCQ: 56% males; SC: 53% males	HCQ 1200 mg/d for 3d then 800 mg/d for 14–21 d	11 to 29 February	Rate of viral negative conversion at 28 days	(56/75 (74.6%) in SC and 53/75 (70.6%) in HCQ) negatively converted before 28 days
	RCT					SC			
Carlucci^48^	USA	PCR positive COVID-19 patients	931 Zinc + HCQ + AZM: 411; HCQ + AZM: 521	Zinc + HCQ + AZ: 63.19 ± 15.18; HCQ + AZ: 61.83 ± 15.97	Zinc + HCQ + AZM: 64.3% males; HCQ + AZ: 61.4% males	HCQ 400 mg/d for 1 d then 200 mg bid for 5d + AZ 500 mg/d for 5 d + zinc sulfate 220 mg bid for 5 d	2 March 2020–5 April 2020	Effect of adding zinc to HCQ and AZM	The addition of zinc sulfate did not impact the length of hospitalization, duration of ventilation, or ICU duration
	Retrospective observational					The same dose as in the other group but without zinc			
Singh^49^	USA	Confirmed COVID-19 patients	1820 (910/910)	HCQ: 62.17 ± 16.81; SC: 62.55 ± 17.62)	HCQ: 53.96% males; SC: 54.94% males	HCQ (dose not mentioned)	20 January, 2020–1 May, 2020	Mortality 30-day and need for mechanical ventilation	Mortality: 11.34% (HCQ) and 11.98% (SC)
	Retrospective cohort					SC			Mechanical ventilation: 5.05% (HCQ) and 6.26% (SC)
Singh^49^	USA	Confirmed COVID-19 patients	1402 (701/701)	–	–	HCQ + AZM (dose not mentioned)	20 January, 2020, to 1 May, 2020	Mortality 30-day and Need for mechanical ventilation	Mortality: 12.27% (HCQ + AZM) and 10.27% (SC)
	Retrospective cohort			–	–	SC			Mechanical ventilation: 5.71% (HCQ + AZM) and 5.85% (SC)
Regina^50^	Switzerland	laboratory confirmed SARS-CoV-2 patients	200	70.0 (55.0–81.0)	60% males		From March 1 to March 25, 2020	Need for mechanical ventilation (MV) at day 14	HCQ: (31.2%); Remdisivir: (100%); Protease inbititors: (31%); Tocilizumab: (82%)
	Retrospective cohort								
Membrillo^51^	Spain	laboratory-confirmed SARS-CoV-2 patients	166 (123/43); 83 patients had a mild clinical picture at admission, 48 moderate, and 35 severe	HCQ: 61.5 ± 16.2; SC: 68.7 ± 18.8	HCQ: 61.8% males; Non HCQ: 62.8% males	Loading dose of HCQ 800 mg + 400 mg, followed by maintenance dose of 400 mg/d	N/A	Mortality	48.8% of patients not treated with HCQ died versus 22% in the group of HCQ (*P* = 0.002)
	Observational Cohort					SC			HCQ increased the mean cumulative survival in the mild-moderate and severe group to 1.8, 1.4, 1.6 times respectively but the difference was statistically significant in the mild group
Lee^52^	South Korea	Confirmed COVID-19 patients	72 (LPV/r: 45 HCQ: 27	Median (IQR); LPV/r: 39 (24–56); HCQ: 37 (24–53)	LPV/r: 44.4%; HCQ: 44.4%	LPV/r: 400/100 mg/d bid	21 Feb 2020 to 21 March 2020	Compare clinical outcomes of both treatments	Disease progression (HCQ) 44% and (LPV/r) 18%
	Retrospective cohort					HCQ: 400 mg/d			
Million^53^	France	PCR positive COVID-19 patients	1061	43.6 ± 15.6	46.4% males	HCQ 200 mg tid for 10 d + AZM 500 mg on day 1 followed by 250 mg/d for 4 d	3 March 2020 to 31 March 2020	Death, clinical worsening, and viral shedding persistence (> 10 days)	91.7% had good clinical outcome and virological cure, 4.4% had viral shedding persistence and 0.75% died
	Retrospective cohort								
Okour^54^	USA	Confirmed COVID-19 patients	36 Patient	Not provided	Not provided	HCQ+/−AZM	March 2020	Probability of negative-PCR in patients	Odds of positive-PCR decrease by 53% for each unit increase in HCQ log-concentration. Similarly, the odds decrease by 61%, and by 12% for each day increase, and azithromycin co-treatment, respectively
	Non-RCT								
Saleh^55^	USA	Confirmed COVID-19 patients	201 CQ: 10; HCQ: 191 (119 patients received AZM in addition to HCQ)	58.5 ± 9.1	57.2% males	CQ 500 mg bid for 1 d then 500 mg/d for 4 d or HCQ 400 mg bid for 1 d then 200 mg bid for 4 d	1–23 March 2020	Assess QT prolongation resulting in Torsade de pointes	440.6 ± 24.9 ms (HCQ/CQ) and 439.9 ± 24.7 ms (HCQ/CQ + AZM) (*P* = 0.834)
	Prospective cohort					The same doses as before + AZM 500 mg/d for 5 days			
Chorin^56^	USA	COVID-19 patients	251 patients	64 ± 13	75% males	HCQ 400 mg bid for 1 d then; 200 mg bid for 4 days + AZM; 500 mg/d for 5 d	–	Assess the change in QTc	QTc > 500 ms, occurred in 23% of patients
	Retrospective cohort								

*aHR* adjusted hazard ratio, *ARDS* acute respiratory distress syndrome; *AZM* azithromycin, *CI* confidence interval, *CQ* chloroquine, *CT* computed tomography, *HCQ* hydroxychloroquine, *ICU* intensive care unit, *IQR* interquartile range, *LPV/r* Lopinavir/ritonavir, *ms* milliseconds, *N/A* not applicable, *PCR* Polymerase chain reaction, *QTc* corrected QT interval, *RCT* randomized control trial, *SC* standard care.

^a^Age was presented either as mean ± Standard deviation or median (Interquartile range).

### Quality assessment

**Figure 2:**
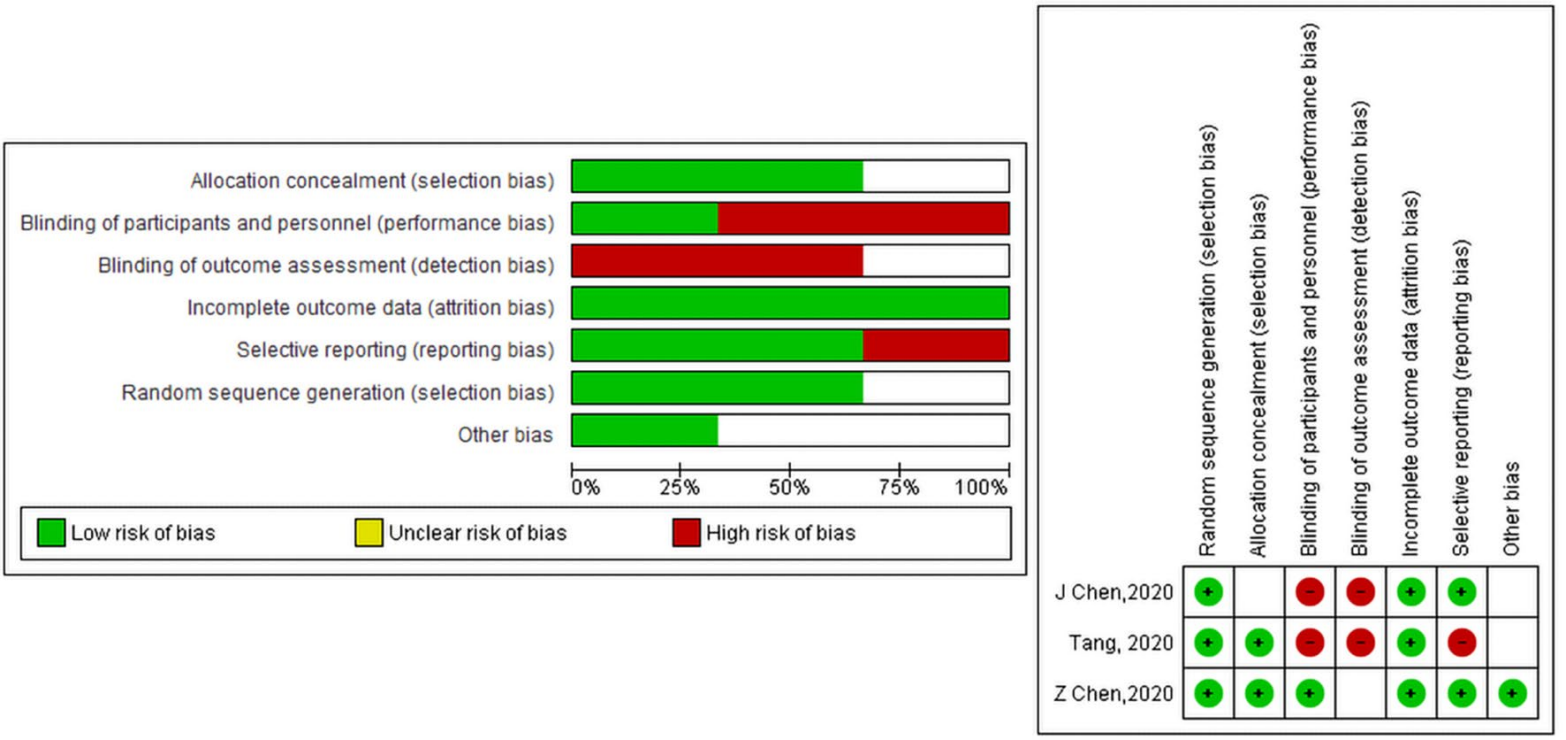
(**a**) Risk of bias graph: review authors' judgements about each risk of bias item presented as percentages across all included studies. (**b**) Risk of bias summary: review authors' judgements about each risk of bias item for each included study.

Quality assessment for the studies included in this meta-analysis was conducted using Cochran risk assesment tool, Jadad, ROBINS-I, and NOS checklists.  Quality assement for RCTs is presented in the summary of the risk of bias graph Fig. 2.

### Publication bias

Publication bias assessment of studied that assessed mortality was conducted by visual inspection of the funnel plot^24^.

### Primary outcome: mortality associated with exposure to CQ/HCQ ± AZM

**Figure 3:**
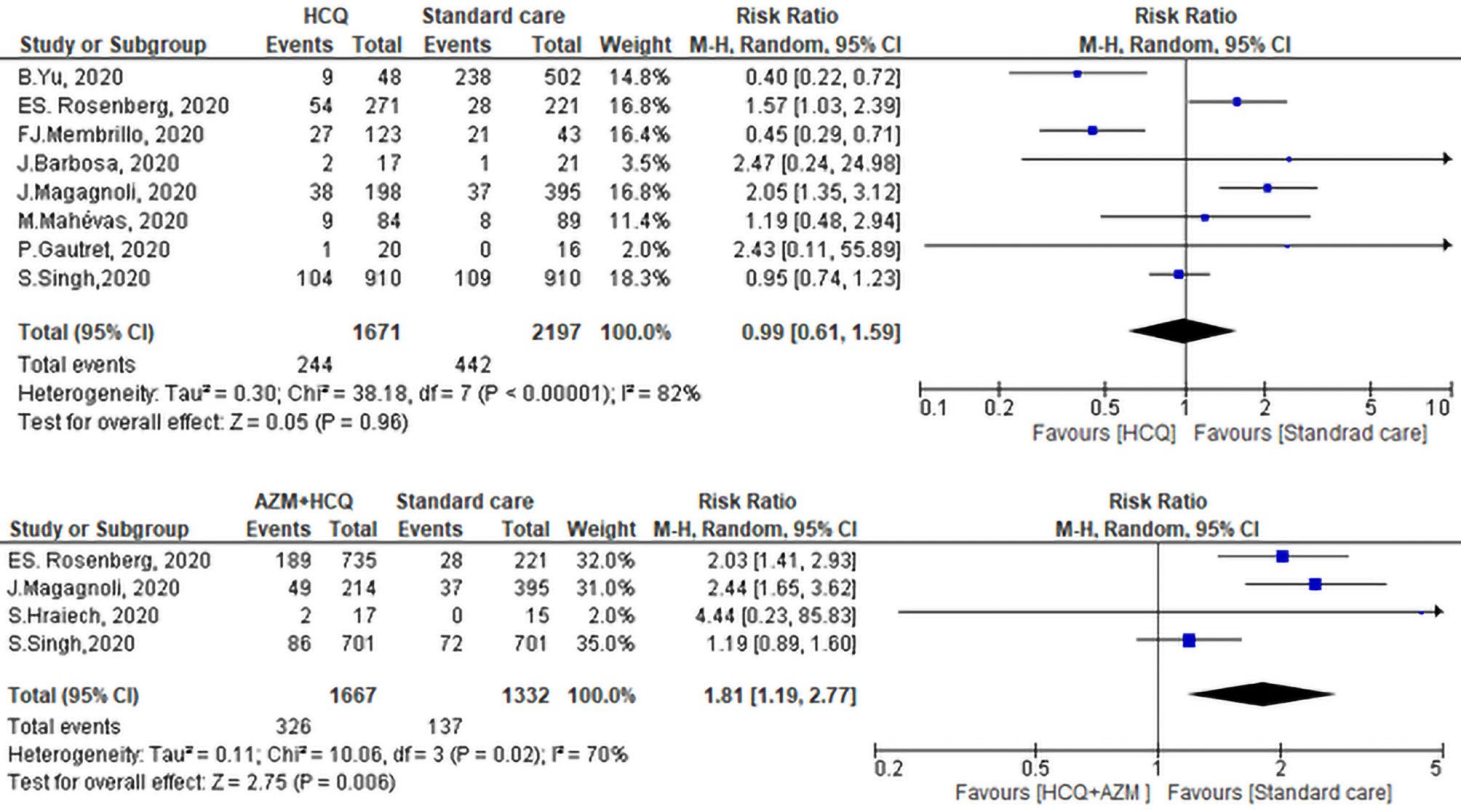
Pooled mortality in the Hydroxychloroquine ± Azithromycin groups versus standard care group.

**Figure 4:**
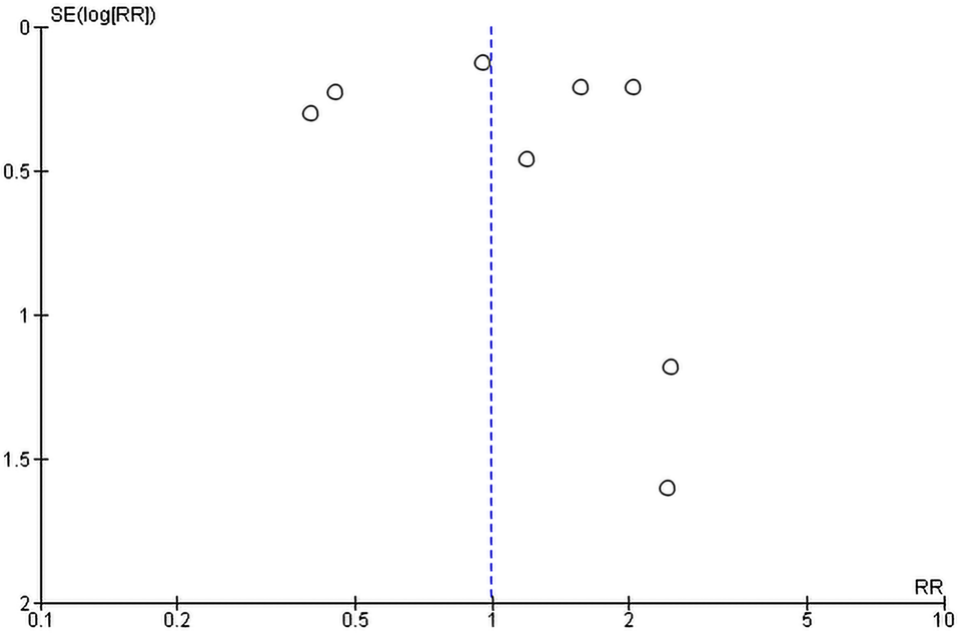
Funnel plot of included studies highlighted the mortality of the Hydroxychloroquine regimen.

Mortality of HCQ was addressed in 8 studies, however, controversial results were seen. Yu et al.^39^ and Membrillo et al.^51^ showed that there was   higher mortality rate in the standard care (SC) group compared to the HCQ group. While Rosenberg et al. and Magagnoli et al.^41^, showed that there was a higher mortality rate among those who did receive HCQ. Of note, among the 20 patients included in the study of Guatret et al.^15^, 6 patients were on AZM. Pooled RR showed that there was no significant difference between the two groups in mortality (RR of 0.99, 95% CI 0.61–1.59, *P* = 0.96, I^2^ = 82%) (Fig. 3). Leave one out sensitivity analysis revealed a considerable heterogeneity at all stages of the test. All studies nearly equally contributed to the overall heterogeneity. Hence, meta-regression was conducted to underline the possible effect of covariates. The risk of mortality was regressed considering mean or median age, country, percentage of male patients, and severity of illness as regressors. Age was not a significant predictor (*P* = 0.323) as the mean or median age was above 60 years across all selected studies except for the study of Gautret, et al.^15^, in which the median age of the participant was 45 years. Moreover, the severity of illness was not significant (*P* = 0.105) as the patients in almost all selected studies were hospitalized with varied clinical status except for the study of Yu et al.^39^, in which patients were all critically ill. Interestingly, the country of the study was a significant predictor for the risk of mortality at two levels (France and USA) setting for China (Yu 2020) as a reference country. Switching from Chinese to French studies increased the relative risk of mortality in HCQ groups by 7.28 times (*P* = 0.001). Similarly, switching from Chinese to American studies increased the relative risk of mortality in HCQ groups by 4.29 times (*P* = 0.005). By Meta-regression, the overall heterogeneity of selected studies was not significant (*P* = 0.243, I^2^ = 22%). The publication bias of the included studies is presented in Fig. 4.

Mortality  among patients treated with HCQ + AZM was compared with those received SC in 4 studies. Pooled analysis revealed that there was no significant difference in mortality between the two groups (RR of 1.81, 95% CI 1.19–2.77, I^2^ = 70%) (Fig. 3). So, we performed leave one out sensitivity test. The study of Singh et al.^49^ contributed most to the overall heterogeneity. By exclusion of this study, the heterogeneity between other studies was insignificant (*P* = 0.71, I2 = 0%), and the overall effect remained significant (RR: 2.23, 95% CI 1.70–2.91, *P* < 0.0001).

### Secondary outcomes

#### Duration of Hospital Stay associated with exposure to CQ/HCQ ± AZM

**Figure 5:**
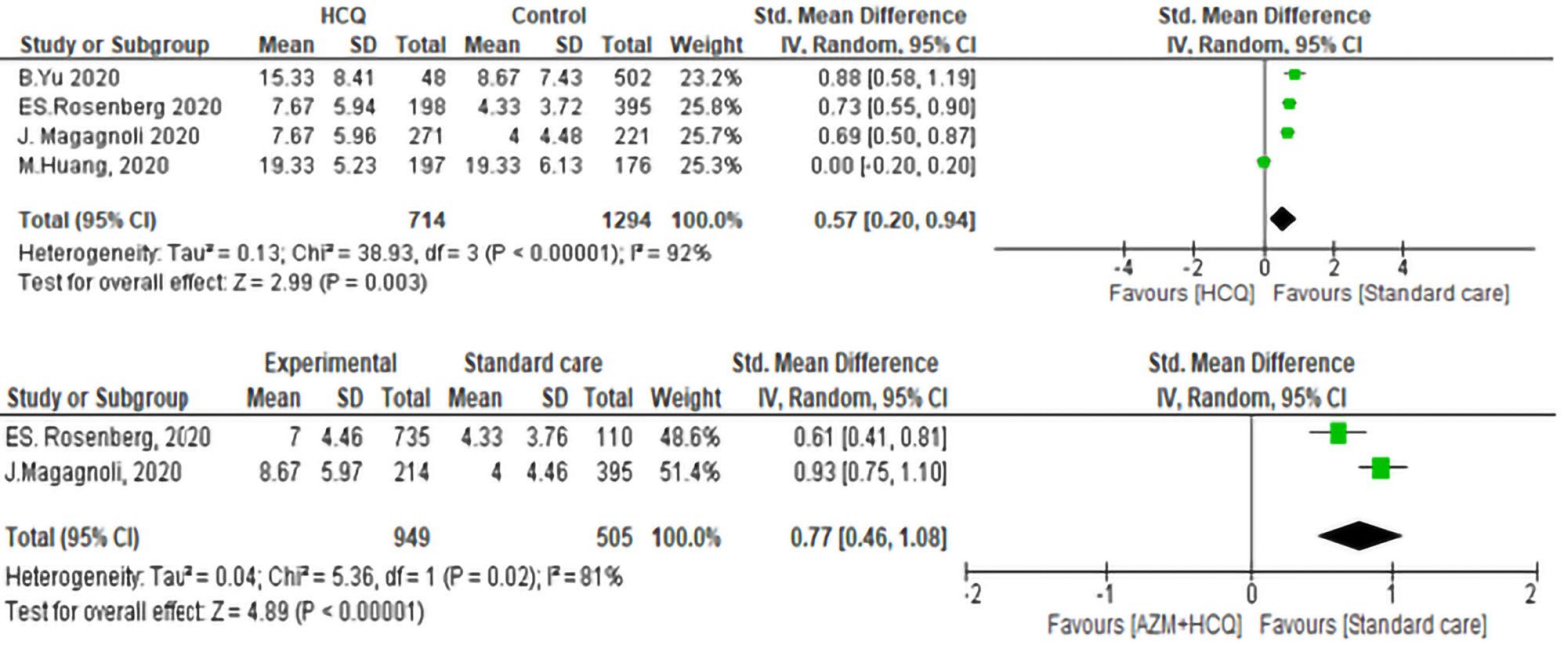
Duration of hospital stay of  Hydroxychloroquine ± Azithromycin versus standard care.

The duration of the hospital stay of patients on the SC group was significantly shorter than the HCQ/CQ group (std. mean difference was 0.57, 95% CI 0.20–0.94, *P* < 0.01). Of the four included studies, three studies favored the SC with std. mean difference ranging from (0.50–1.19). The heterogeneity of the included studies was as follows (I^2^ = 81%, *P* < 0.01) (Fig. 5). In the sensitivity analysis, the study of Huang et al.^40^, contributed most to heterogeneity. Excluding this study made the overall effect relatively higher (std. mean difference = 0.73, 95% CI 0.62–0.85, *P* < 0.01) and the test of heterogeneity was not significant (*P* = 0.54, I^2^ = 0%).

The duration of hospital stay in case of treatment with (HCQ + AZM) combination versus SC was reported in two studies. In the analysis, we found a significant difference between both groups. Patients treated with HCQ + AZM combination had longer mean duration of hospital stay than the SC group. The pooled std. mean was 0.77, 95% CI 0.46–1.08, *P* < 0.01. The heterogeneity was statistically significant, *P* < 0.01, I^2^ = 92% (Fig. 5).

#### Virological Cure associated with exposure to CQ/HCQ ± AZM

**Figure 6:**
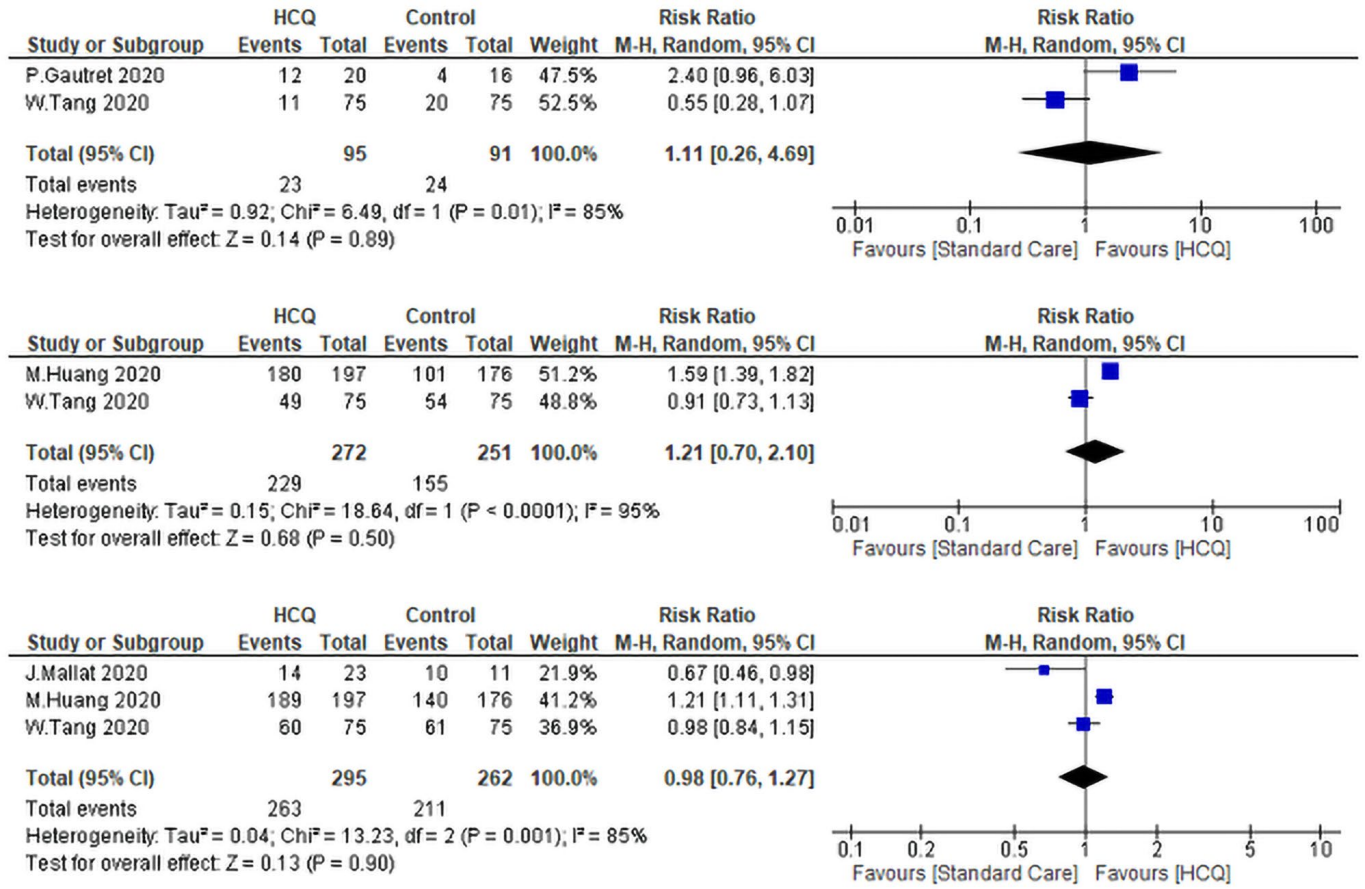
Forest plot for pooling risk ratios regarding virological cure rate on day four, 10 and 14 respectively.

**Figure 7:**
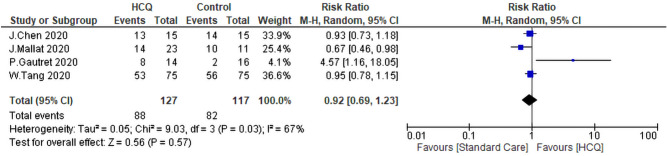
Forest plot for pooling risk ratios regarding the overall virological cure rate.

To get more insight over the virological cure rate (VQR), we were able to find two studies that analyzed the VQR of HCQ on day 4, two studies analyzed it on day 10, and three analyzed it on day 14. There were no differences between the HCQ group and the SC group [(RR: 1.11, 95% CI 0.26–4.69), (RR: 1.21, 95% CI 0.70–2.10), and (RR: 0.98, 95% CI 0.76–1.27) (Fig. 6). The heterogeneity of the three analyses were (I^2^ = 85%, *P* = 0.01, I^2^ = 95%, *P* < 0.01, and I^2^ = 85%, *P* < 0.01) respectively. The comparison of the VQR at day 14 was subjected to leave one out sensitivity analysis. There was substantial heterogeneity between studies at all stages of the test. However, the study of Huang et al.^40^ contributed to most of the heterogeneity. The overall VQR was not statistically significant between the intervention group and the SC. The pooled RR was 0.92, 95% CI = 0.69–1.23, *P* = 0.57. The heterogeneity of the studies was as follow I^2^ = 67%, *P* = 0.03 (Fig. 7). Sensitivity analysis revealed that the study of Gautret et al.^30^ contributed to most of the heterogeneity. By exclusion of this study, the heterogeneity was not significant between the rest of the studies (*P* = 0.26, I^2^ = 25%).

**Figure 8:**
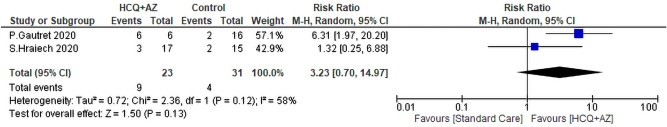
The virological cure rate of Hydroxychloroquineand azithromycin versus standard care.

Two studies reported the virological cure of HCQ + AZM in combination versus SC. The archived VQR of HCQ + AZM (9/23) did not differ significantly from the SC (4/31) (RR = 3.24, 95% CI 0.71–14.74, *P* = 0.13). The heterogeneity of the study was not significant *P* = 0.12 I^2^ = 58% (Fig. 8).

#### Need for mechanical ventilation

**Figure 9:**
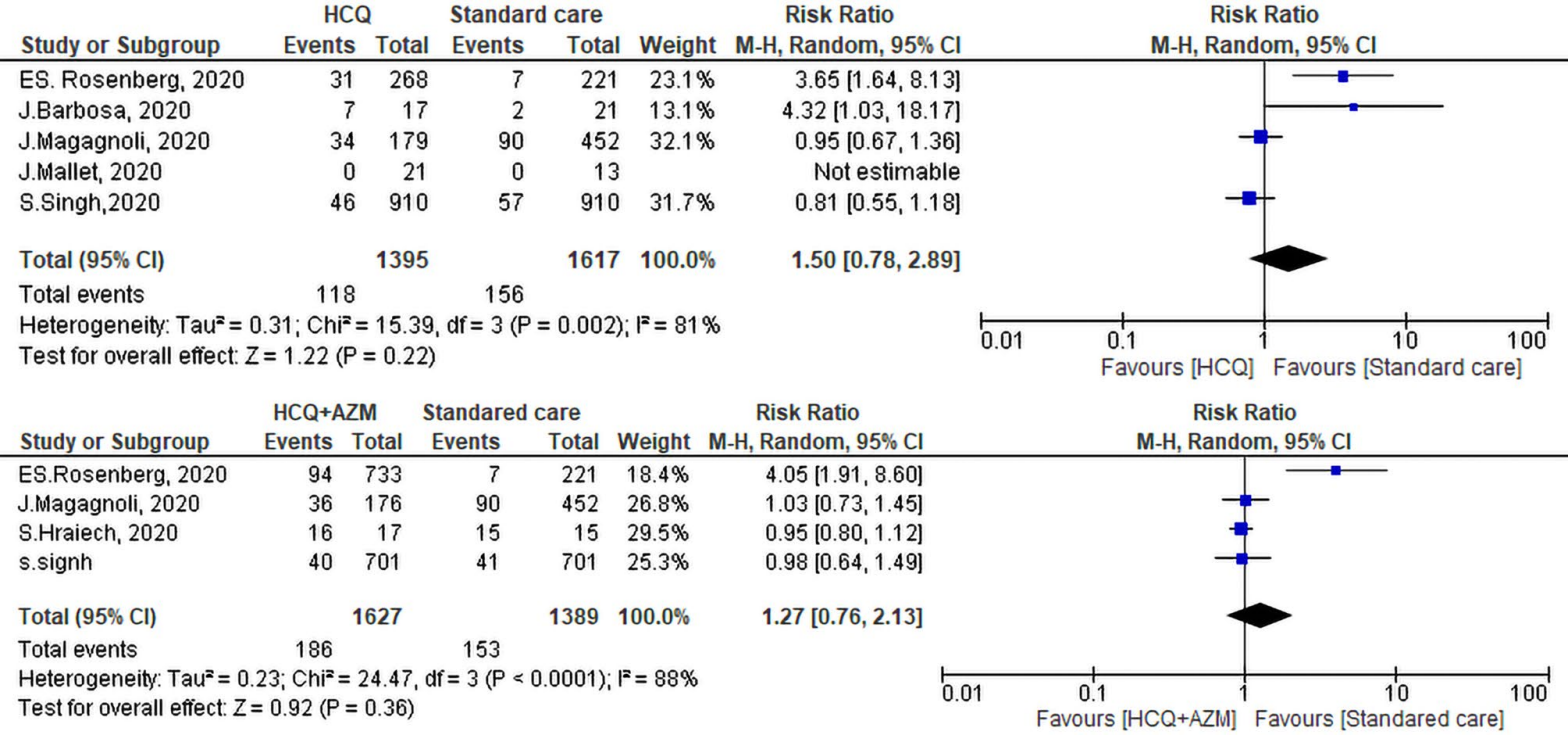
Need for mechanical ventilation of the Hydroxychloroquine ± Azithromycin versus standard care.

The need for MV was reported in five studies; 118 of 1395 patients on HCQ required MV versus 156 of 1617 patients on SC. Two studies reported more need for MV among the SC group, the range of individual RR is 1.03–18.17, 95% CI. Nonetheless, analysis, revealed that there was no significant difference between both groups in the need for MV (RR = 1.50, 95% CI 0.78–2.89, *P* = 0.22). The test of heterogeneity was statistically significant I^2^ = 81%, *P* = 0.001. Upon performing leave one out sensitivity analysis, there was a substantial heterogeneity at all stages of the test. In fact, the study of Rosenberg et al.^35^ contributed most to heterogeneity between studies. By exclusion of this study, the heterogeneity  became insignificant (*P* = 0.08, I^2^ = 61%) while the overall effect remained insignificant (*P* = 0.95). Pooled analysis of the four studies that evaluated the need for MV among (HCQ + AZM) and SC groups revealed that 186 of 1627 patients on HCQ + AZM required MV versus 153 of 1389 patients  on the SC. This difference was not significant between both groups (RR = 1.27, 95% CI 0.76–2.13, *P* = 0.36). The heterogeneity of the studies was as follows I^2^ = 88%, *P* < 0.01 (Fig. 9). Leave one out sensitivity analysis was performed. Rosenberg et al.^35^  study contributed most to overall heterogeneity. By exclusion of this study, the heterogeneity between other studies was insignificant (*P* = 0.82, I^2^ = 0%) while the overall effect remained insignificant (RR: 0.96, 95% CI 0.84, 1.11, *P* = 0.59).

#### Time to negative PCR associated with exposure to CQ/HCQ

**Figure 10:**
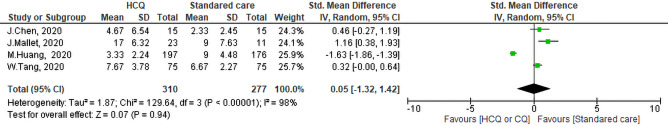
Forest plot for pooling std. mean differences of the time to a negative polymerase chain reaction.

Four studies evaluated the time to negative PCR after administration of CQ/HCQ. One study proved that intervention was more effective (Std. mean difference = −1.63, 95% CI − 1.86 to − 1.39), however, the pooled Std. mean difference of these studies indicated that there was no significant differences between the HCQ group and the SC group in terms of the time for PCR to turn negative (Std.mean = 0.05, 95% CI − 1.32 to 1.42, *P* = 0.94). The measured heterogeneity was statistically significant I^2^ = 98%, *P* < 0.01 (Fig. 10). Sensitivity analysis revealed that the study of Huang et al.^40^ contributed most to heterogeneity. By the Exclusion of this study, the heterogeneity between the rest of the studies was insignificant (*P* = 0.45, I^2^ = 48%). Moreover, pooled std. mean turned to be significantly shorter in SC groups (std. mean difference = 0.55, 95% CI 0.09, 1.02, *P* = 0.02).

#### Radiological improvement

**Figure 11:**
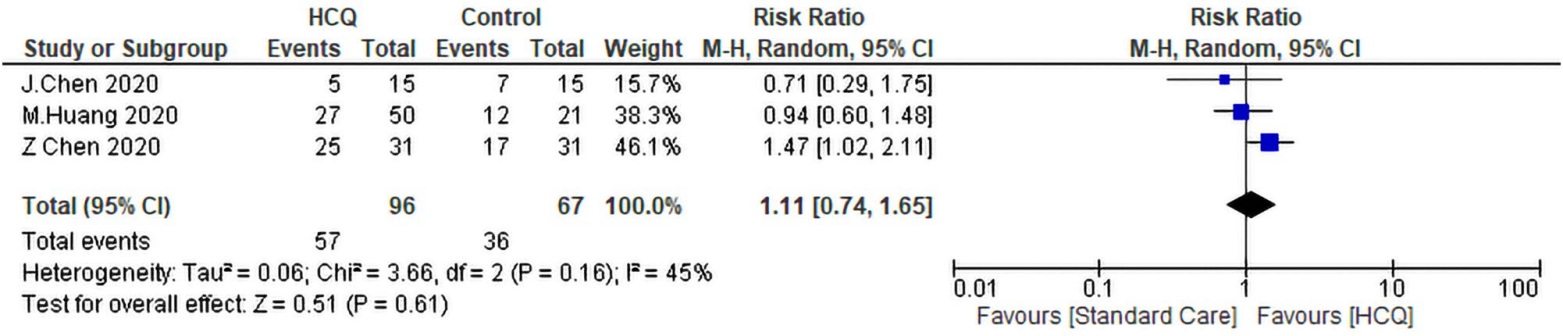
Forest plot for pooling risk ratios regarding radiological improvement.

Three studies evaluated the diference in radiological improvement between the HCQ group and the SC group. Only one study reported better radiological improvement of the SC (RR = 1.47, 95% CI 1.02–2.11). The pooled analysis revealed that treatment of COVID-19 patients with  HCQ didn’t improve the radiological findings signficantly compared to SC (RR: 1.11, 95% CI 0.74–1.65, *P* = 0.61) (Fig. 13). The heterogeneity between studies was not significant (*P* = 0.16, I^2^ = 45%) (Fig. 11).

Sensitivity analysis showed that Chen^28^ contributes most of the heterogeneity. By exclusion of this study, the I^2^ index approached 0%, while the overall effect remained insignificant (RR: 0.89, 95% CI 0.60–1.34).

#### Side effects

**Figure 12:**
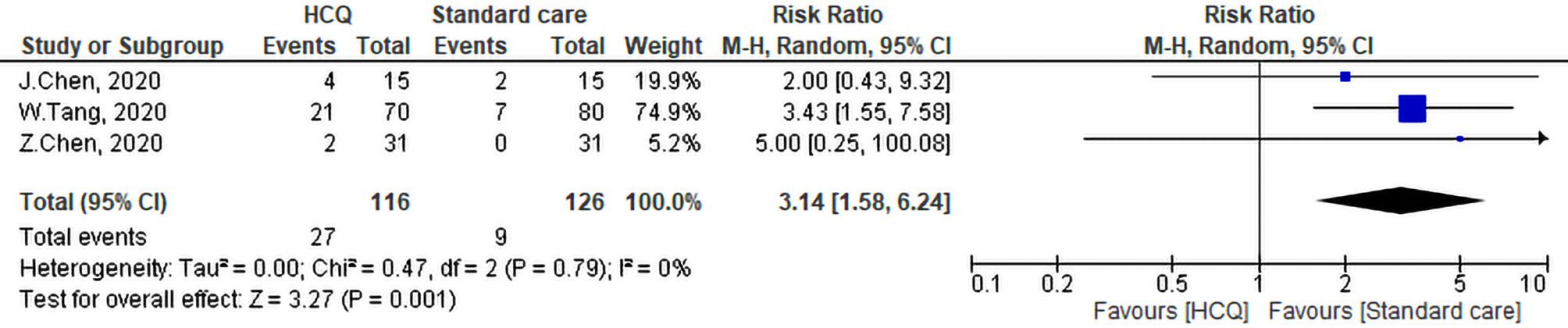
Side effects of the Hydroxychloroquine versus the standard care.

The three studies that addressed side effect of HCQ revealed that intervention group witnessed greater side effects than the SC (27/116) and (9/126) respectively, this difference was statistically significant (pooled RR = 3.14, 95% CI 1.58–6.24, *P* < 0.01) (Fig. 12). The heterogeneity of the studies was not significant (I^2^ = 0%, *P* = 0.79).

#### Worsening of clinical symptoms

**Figure 13:**
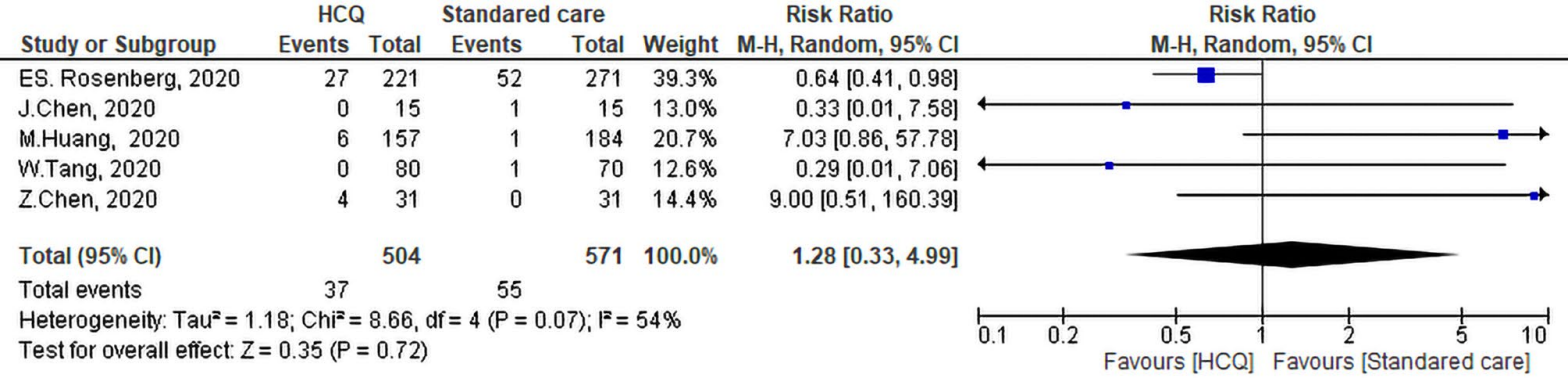
Clinical worsening of the Hydroxychloroquine versus the standard care

Five studies evaluated the differences between the HCQ or CQ group and the SC group in terms of clinical worsening. The meta-analysis showed that there was no differences between the HCQ group and the SC group regarding the worsening of clinical symptoms (RR: 1.28, 95% CI 0.33–4.99, *P* = 0.72). The heterogeneity of the studies was not significant *P* = 0.07 and I^2^ = 54% (Fig. 13). Sensitivity analysis revealed that the study of Huang et al.^40^ contributed most to this heterogeneity. By exclusion of this study, the heterogeneity of studies was not significant (*P* = 0.29, I^2^ = 19%) while the overall effect remained insignificant (RR: 0.74, 95% CI 0.28–1.97, *P* = 0.72).

### Other outcomes

#### QT prolongation

Many studies have evaluated the effect of HCQ on inducing QT prolongation; Chong et al.^29^ demonstrated that 45.5% of patients exposed to HCQ developed QT prolongation. On the same vein, Broek et al.^37^ noticed that 23% of CQ patients developed significant QT prolongation (˃500msec). Voisin et al.^38^ reported that of 50 patients treated with HCQ + AZM; 6 patients stopped the treatment due to significant QT prolongation, and 38 patients (76%) presented with short-term modifications of QTc duration (meaning > 30 ms). The same figure was reported by Ramireddy et al.^42^ and Saleh et al.^55^ who reported that there was no difference regarding QT prolongation in-between patients treated with CQ or HCQ. Moreover, combination with AZM increased the risk of QT prolongation as (470.4 ± 45.0 ms) versus monotherapy (453.3 ± 37.0 ms), *P* = 0.004. This increase in QT prolongation was incriminated in discontinuation of treatment in 3.5% of the studied patients. On the other hand, Rosenberg et al.^35^ reported a lower incidence of QT prolongation among patients treated with a combination of HCQ + AZM than HCQ alone (11.0% vs. 14.4%) respectively. Finally, Chang et al.^57^ reported that 17.9% of patients treated with HCQ ± AZM had QT prolongation > 500 m-second. The prolongation of QT after administration of HCQ + AZM or HCQ alone was not statistically significantly different.

#### Fever

A total of three studies that evaluated body temperature normalization after HCQ therapy; Huang et al.^40^ reported that body temperature returned normal after a geometric mean (coefficient variation), 1.2 (53.5) among HCQ group versus 1.9 (110.0) among non HCQ (*P* = 0.0029), while Chen et al.^14^ reported that patients’ temperatures returned to normal at approximately at the same rate in both groups. (Median 1, IQR 0–2 for HCQ and Median 1, IQR 0–3 for no-HQR). Chen et al.^28^ reported that the duration of fever was shorter in the HCQ group (mean 2.0 ± 0.2) than in the non-HCQ group (mean 3.1 ± 1.5).

#### Cough

Chen et al.^28^ reported that 15 of 31 (48.39%) of the control patients and 22 of 31 (70.97%) intervention patients had reported cough resolution. This difference was statistically significant *P* = 0.0016.

#### Laboratory test improvement

Two studies evaluated the change in laboratory test after exposure to HCQ, first Mallat et al.^44^ reported that median lymphocyte count at day 7 was 1870 (1115–2625) compared to its baseline 1890 (1430–2230) in the control group, while it was 1650 (980–1950) at day 7 in the intervention group compared to its baseline level 1650 (980–1950). Additionally, the median serum ferritin level at day 7 was 398 (52–1030) compared to its baseline 292 (33–1085) in the control group, while it was 249 (130–614) at day 7 in the intervention group compared to its baseline level 165 (63–320). Barbosa et al.^43^ reported that change in neutrophil to lymphocyte ratio was higher in HCQ (9.55 ± 21.5) than SC (1.58 ± 6.26) but this increase was not significant. Similarly, the change in absolute lymphocyte count was not statistically significant between both groups (− 0.61 ± 0.52 of HCQ group vs. − 0.61 ± 0.38 SC).

## Discussion

COVID-19 is a life-threatening disease with no proven effective therapy. There are a few high-quality randomized control trials that evaluated the effectiveness of CQ/HCQ in the management of COVID-19. This attempt was followed by many reported poor methodologically designed observation studies that addressed the role of these drugs alone or in combination with AZM in facing this pandemic. Of note, most of these studies recruited few heterogeneous numbers of participants aimed at studying different outcomes with variable endpoints providing different doses of drugs for different durations.

In this study, we shed the light on the effectiveness and safety of CQ/HCQ with or without AZM in the management of COVID-19 to provide robust evidence for health policy decision-makers to face the ongoing pandemic. We included 14 articles to study the desired outcomes. The included studies according to their design were three RCT, two non-CT, three case-control, and six retro or prospective cohort. All included studies were conducted at hospitals. In the systematic review section, the highest number of recruited patients in a single study was 1438^35^, while the smallest number was 11 subjects^29^. The outcome that included the highest number of patients in pooled outcome analysis was mortality (3868 patients), while the smallest number of patients was included in studying the effectiveness of HCQ and AZM in achieving virologic cure (54 patients).

Based on the finding of this meta-analysis, mortality of the HCQ group was not different from that of SC. The country of residency was a significant predictor of mortality outcomes. The alarming finding was that SC patients had lower numbers of mortalities if they were compared to the AZM and HCQ combination group. Exposure to HCQ was associated with a longer duration of hospital stay, whether AZM was included or not in the treatment regimen. Generally, exposure to HCQ alone or in combination with AZM was not associated with any witnessed decrease in the need for mechanical ventilation. Regarding the difference in viral clearance between HCQ and SC, the time to negative conversion was not statistically different between the two groups (HCQ and SC). Similarly, virologic cure rates at either day 4, day 10, or day 14were not different between both groups. It is worthy to mention that adding AZM to HCQ did not affect the cure rate compared to SC. Furthermore, neither clinical worsening nor radiological improvement of the studied patients was affected concurrently with exposure to HCQ. Side effects were more encountered if patients were treated with HCQ/CQ.

### Mortality HCQ and AZM

In the current research, mortality rate of HCQ alone did not significantly differ from that of the SC. Due to high heterogeneity and failure of sensitivity analysis to identify the source of heterogeneity, we carried out meta-regression analysis. In Meta-regression analysis, the heterogeneity dropped to 22% and we identified that country was a strong predictor of mortality. The insignificant difference in mortality after exposure to HCQ was similar to what was reported by Shamshirian et al.^58^ who almost included the same studies in their meta-analysis with a high heterogeneity of 86.93%. However, in their meta-regression analysis age was the only significant predictor of mortality concurrently with exposure to HCQ. We speculate that not only receiving HCQ is the only predictor of mortality, but also access, quality, and availability of health services within different countries are considered as a strong predictors of disease prognosis^59^. However, a country like France that provides one of the best health services worldwide, had witnessed the second-highest number of case-fatality ratio due to COVID-19 (5.9%) after Mexico (10.5%). On the other hand, a country like Morocco which is ranked 29th on the list of the world health system had a case fatality ratio of 1.8%^60,61^. These discrepancies in case fatality ratio despite the difference in health service quality may trigger the need for studying other determinants of disease outcome  in different countries like ethnicity, population age structure^62^, national interventional measures (i.e. lockdown strategies), PCR testing capabilities^63^, the bias in a testing (more diseased individuals are priority) cultural habits, viral strains, and history of previous vaccinations (BCG vaccine)^64^. Notably, one of the alarming findings was that mortality of the HCQ and AZM was significantly higher than SC (pooled RR = 1.81, 95% CI 1.19–2.77). This significant risk ratio remained even after excluding a study that had a high risk of bias. The increased mortality of this combination may be due to the increased risk of arrhythmia and cardiac arrest. The arrhythmogenic effect of HCQ is due to its structural similarity with quinidine (Class 1A antiarrhythmic drug.) This group inhibits voltage-gated sodium and potassium channels resulting in QT prolongation and increased risk of torsade de point^65^. Mercuro et al.^66^hypothesized that the combination of HCQ and AZM (53 patients) versus HCQ alone (37 patients) can increase the risk of QT prolongation (23.0 vs. 5.5 ms). It is worthy to mention that a randomized control trial adopted a high dose of HCQ and AZM was suspended due to severe cardiotoxicity^25^. On the same line, Shamshirian et al.^58^ declared that mortality among patients received the regimen of HCQ + AZM was 3.5 times higher than the SC. This risk ratio was nearly the double value of ours (1.81), we speculate that this difference is due to the inclusion of two additional researches; the study of Hraiech^32^, and Singh^49^. They contributed to 37% of the weight of the outcome and did not prove higher mortality among patients who received HCQ and AZM. Similarly, Kashour et al.^67^ reported a 1.32 increased in RR of mortality among patients on HCQ + AZM. They included four studies, the highest RR was reported by Kuderer et al.^68^ (2.93) and contributed to 14.64 of the overall weight of the study, however, this study included patient with active or history of malignancy. This co-morbidity might contribute to the high reported RR.

### Duration of hospital stay of HCQ ± AZM versus standard care

Treating COVID-19 patients with either CQ/HCQ alone or in combination with AZM did not significantly shorten the duration of hospital stay. Patients on the SC stayed shorter in the hospital either if they were compared to patients received CQ/HCQ (standard mean difference 0.54, 95% CI 0.20–0.94) or HCQ + AZM (standard mean difference 0.77, 95% CI 0.46–1.08). The reported heterogeneity dropped to 0% if the study of Huang et al.^40^ was removed from the pooled analysis. Absolutely, this increase in the duration of hospital stay can increase the burden on health care facilities and health care workers, decrease the availability of places for new admissions, and increase the burden on patients themselves especially in low-middle income countries with devastated health system. It is important to note that, a recently published systematic review of 52 studies concluded that duration of hospital stay and intensive care admission were also affected by other factors like time of epidemic and country of citizenship regardless the treatment protocol. Patients living in China had longer duration of hospital stay than other countries^69^.

### Virological cure rate of CQ/HCQ ± AZM versus standard care

In vitro study showed that HCQ and CQ were effective in inhibiting the growth of different viruses including SARS corona, enteroviruses, ebola, and Zika virus, however, results of in vivo studies were less promising. Historically, it was very effective in achieving higher virological response if combined with pegylated interferon and ribavirin in treatment of hepatitis C virus infection. In addition to the aforementioned advantages, these drugs are relatively safe, cheap, and worldwide available. These facts encouraged researchers to study the effectiveness of HCQ/CQ in the treatment of COVID-19^70^. In the current research, the achieved cure rate of HCQ (day 4, 10, and 14), and time to negative conversion among the HCQ group were not statistically different from the SC. It is worthy to mention that the term SC was not firmly defined in each study; this may represent a source of prescription bias. Moreover, doses and duration of treatment with either CQ or HCQ were not the same across the different included studies considering the wide rang of half-maximal effective concentration (EC50) of both agents^71^. Another explanation is that HCQ is associated with impairment of interferon alpha and gamma production resulting in impaired immune response^72^. Interestingly, the pooled standard mean difference of this outcome included the paper published by Huang et al.^40^, resulted in a significant heterogeneity in many outcomes especially time till virologic cure. By reviewing this article, we found that the authors did not address the effectiveness of this combination versus SC^41^.

### Need for mechanical ventilation of AZM + HCQ versus standard care

Of COVID-19 patients, about 5–15% need intensive care surveillance any ventilatory support. Deaths among mechanically ventilated patients ranged from 20.5^73^ to 100%^74^. This high fatality rate among ventilated patients may be due to the non-classical acute respiratory syndrome caused by COVID-19^75^. Thus we aimed at studying the need for mechanical ventilation among patients who received either CQ or HCQ alone or in combination with AZM. In our study, we included 5 studies in the comparison of HCQ with SC  and 4 studies in comparing (HCQ + AZM) with SC. Both analyses revealed that using HCQ either alone or in combination with AZM for treatment of COVID-19 did not reduce the need for MV. Of note, our results are in agreement with Shamshirian et al.^58^ who published an MA of two studies addressing the need for MV among HCQ and SC.

### Fever and cough

The initial manifestation of COVID-19 are fatigue, low-grade intermittent fever of prolonged duration, myalgia, dry cough and shortness of breath, which then either improve spontaneously or with conservative therapy or progresses to dyspnea and productive cough^76^. Resolution of respiratory symptoms and fever is one of the symptoms-based indicators of disease recovery. In this study, we evaluated the recovery of these two symptoms after exposure to HCQ^77^. Huang et al.^40^ and Chen et al.^28^ demonstrated that patients treated with CQ recover from fever faster than those on SC, however, Chen et al.^27^ did not report any significant difference between both groups in resolution of fever. Chen et al.^28^ reported a more significant resolution of cough among patients exposed to HCQ. Due to insufficient data, we did not conduct a meta-analysis.

### Radiological improvement and clinical worsening

Computed tomography is more sensitive tool than X-ray in diagnosis of COVID-19. The predominant radiological feature of COVID-19 patients is ground-glass opacification, consolidation, air bronchogram, and nodular opacities without pleural effusion. Moreover, it can be used to follow the disease course^78,79^. In this metanalysis, treatment with HCQ did not provide any additional benefit in terms of radiological improvement or clinical worsening versus the SC. We included three published articles in this analysis of the impact of HCQ on radiological improvement and five articles evaluated the effect of CQ/HCQ on clinical worsening. The heterogeneity of both analyses was 45% and 54% respectively.

### QT prolongation

Abnormal myocardial repolarization results in QT interval prolongation. The normal QT interval is 450 ms in females, and 460 ms in males^80^. Among patients treated with HCQ, QT prolongation was identified in 23–45.5%^29,37^. About 12% of patients on AZM and HCQ stopped treatment due to significant QT prolongation. It is important to notice that there was no difference in the incidence of drug-induced QT prolongation by CQ or HCQ. A combination of HCQ and AZM increases the risk, however, Rosenberg et al.^35^ reported a lower incidence of QT prolongation among patients who received this combination versus SC.

### Side effects

Chloroquine is used as a chemoprophylactic and therapeutic agent for malaria and amebiasis^81^, while HCQ is a less toxic metabolite of chloroquine used to treat SLE, RA, etc.^82^. Nonetheless, these agents can cause ocular manifestation starting from blurring of vision up to optic disc pallor in the end-stage^83^. In this meta-analysis, we included three published pieces of research that addressed the reported side effects of HCQ treatment. Patients on HCQ treatment had a higher risk of experiencing side effects, (pooled RR = 3.14, 95% CI 1.58–6.24) with I^2^ of 0%. In this work, the reported side effects were diarrhea, headache, rash, elevated transaminases, fatigue, and anemia.

### Limitation

Our analysis must be interpreted in the context of the limitations of the available data; despite the huge number of published articles during the COVID-19 pandemic, many of these studies lack good quality and may contain inconsistent results. There is an urgent need for high-quality randomized control trials that address the effectivness of HCQ.

Consequently, we depended in our analysis on few published or even cited preprints. These numbers may be considered insufficient to provide robust evidence on HCQ/CQ supplementation. Moreover, we included many observational studies due to the scarcity of randomized control trials. It is well established that observational studies cannot discover causality. This fact also contributed to the highly found heterogeneity of analysis especially for the study of Huang et al.^40^. After leave one sensitivity analysis the heterogeneity dropped to acceptable value in many outcomes. Another important source of bias was patient selection bias; as some studies did not classify patients according to their disease’s severity. This source of bias may significantly affect the course of illness. Differences in HCQ and AZM dose, duration of treatment, and route of administration may also affect the consistency of our results. We could not perform a subgroup analysis based on disease severity as there is no gold standard tool that uses clinical features or laboratory parameters to classify different disease severity.

## Conclusion

Treating COVID-19 patients with CQ/HCQ did not decrease mortality. even it was increased   if AZM was added. Besides, CQ/HCQ alone or in combination with AZM increased the duration of hospital stay. Overall virological cure rate and that on days 4, 10, or 14 were not affected by receiving HCQ. Adding AZM to HCQ/CQ did not show any benefit in terms of virological cure as well. The Need for MV was not improved by exposure to CQ/HCQ alone or in combination with AZM. Moreover, CQ/HCQ, did not neither shorten the duration till conversion to negative PCR, prevent radiological progression,  nor affect clinical worsening of the disease. Future randomized clinical trials are needed to confirm these conclusions.
